# Beyond chemoradiotherapy: improving treatment outcomes for patients with stage III unresectable non-small-cell lung cancer through immuno-oncology and durvalumab (Imfinzi®▼, AstraZeneca UK Limited)

**DOI:** 10.1038/s41416-020-01071-5

**Published:** 2020-12-08

**Authors:** Priyanka Patel, Doraid Alrifai, Fiona McDonald, Martin Forster, on behalf of AstraZeneca UK Limited

**Affiliations:** 1 Department of Radiotherapy, The Royal Marsden NHS Foundation Trust, London, UK; 2 Lungs for Living Research Centre, UCL Respiratory, Rayne Institute, University College London, London, UK; 3 University College Hospital, London, UK; 4 UCL Cancer Institute, University College London, London, UK

## Abstract

The treatment paradigm of non-small-cell lung cancer (NSCLC) has rapidly changed in recent years following the introduction of immune-checkpoint inhibition (ICI). Pre-clinically, both chemotherapy and radiotherapy modulate the tumour microenvironment, providing the rationale for clinical trials evaluating their role in combination with immunotherapy. Standard-of-care treatment for patients with unresectable stage III disease is concurrent chemoradiotherapy (cCRT); however, only recently, the combination with ICI has been explored. The Phase 3 PACIFIC study randomised 713 patients with confirmed locally advanced, unresectable, stage III NSCLC, whose disease has not progressed following cCRT, to either the anti-programmed death-ligand 1 (PD-L1) agent durvalumab (Imfinzi^®^▼, AstraZeneca UK Limited) or placebo. Patients with a PD-L1 status ≥1% treated with durvalumab had a significantly longer median progression-free survival compared with placebo (17.2 vs. 5.6 months, respectively; HR: 0.51; 95% CI: 0.41–0.63), prolonged median overall survival (OS) (NR vs. 28.7 months, respectively; HR: 0.68; 99.73% CI: 0.47–0.997; *P* = 0.0025) and long-term clinical benefit (3-year OS HR: 0.69; 95% CI: 0.55–0.86). Grade 3 or 4 toxicity was marginally greater in the durvalumab cohort versus placebo (30.5% vs. 26.1%). Based on these results, durvalumab has been licensed in this setting, and further clinical trials are exploring the use of ICI in unresectable stage III NSCLC.

## The evolving landscape of treatment for advanced non-small-cell lung cancer

Treatment of metastatic non-small-cell lung cancer (NSCLC) has undergone a rapid transformation in a relatively short time. Following the advent of platinum doublet chemotherapy,^1^ treatment advances have been based on an improved biological understanding of lung cancer, delivered through refined pathological and molecular classification. Treatment has evolved to include targeted therapies, such as the addition of anti-angiogenics to chemotherapy and the use of small-molecule inhibitors in patients whose tumours harbour actionable genetic alterations.^2,3^ More recently, immune-checkpoint inhibition (ICI) has shown promise in patients with advanced cancer.^4–6^ Indeed, disrupting the physiological balance between immune system activation and inhibition through receptors on cells such as T lymphocytes has become the cornerstone of modern immunotherapy. Monoclonal antibodies have been shown to suppress co-inhibitory receptors (also known as immune checkpoints) such as cytotoxic T lymphocyte-associated protein 4 (CTLA-4) and programmed cell death-1 (PD-1), resulting in the activation of the immune system and subsequent tumour regression.^7^ As such, immune-checkpoint inhibitors targeting the PD-1/programmed death-ligand 1 (PD-L1) axis have gained global attention in light of positive findings in several landmark studies in advanced NSCLC.^8–14^

### Rationale for combining radiotherapy with immunotherapy

Radiotherapy is a modulator of the immune response and tumour microenvironment; emerging evidence suggests that radiotherapy triggers the patients’ immune system to recognise the increase in T-cell diversity. In brief, local radiotherapy (RT) damages tumour DNA, in particular by causing double-strand DNA breaks, resulting in the release of tumour-associated antigens (TAAs).^15^ Subsequent attempts by damaged cancer cells to undergo mitosis lead to activation of the stimulator of interferon gene (STING) protein, which triggers interferon 1 (IFN-1) production and dendritic cell recruitment.^16^ Activated dendritic cells present TAAs through cross-presentation to CD8 + T cells, which are then activated against the remaining viable tumour cells.^17,18^ This rationale could help support the potential for synergy with anti-PD-L1 treatments, which also stimulate CD8 + T cells to set off a downstream cascade that results in tumour regression.^18^

### Immunotherapy for the treatment of stage III NSCLC

The standard of care for patients with a good performance status and unresectable stage III NSCLC is concurrent chemoradiotherapy (cCRT), which consists of platinum-based doublet chemotherapy delivered during radiotherapy.^19,20^ Several clinical trials support this approach, including the Phase 3 RTOG 9410 study that randomised 610 patients, with a Karnofsky performance status of 70 or greater, to either cCRT or sequential CRT (sCRT), demonstrating a superior survival advantage in patients who received either concurrent cisplatin/vinblastine or cisplatin/etoposide versus sequential cisplatin/vinblastine treatment (*P* = 0.046).^21^ The Phase 3 study of concurrent versus sequential thoracic radiotherapy in combination with mitomycin, vindesine and cisplatin in this patient population reported that concurrent treatment resulted in a significantly increased response rate and improved median overall survival (OS) when compared with sequential treatment.^22^ In support of this, a meta-analysis comparing cCRT with radiotherapy alone also supports the use of cCRT and reported a superior survival advantage for patients receiving cCRT compared with radiotherapy.^23^ Despite the superiority of cCRT over sequential radiotherapy or radiotherapy alone, the median progression-free survival (PFS) among patients who have received cCRT remains poor (~8 months) with survival at 5 years of only ~15%.^24,25^

Further treatment intensification strategies have been explored but have failed to demonstrate a significant OS benefit. Studies evaluating the role of induction or consolidation chemotherapy in patients following CRT have failed to establish meaningful benefit.^24,26^ Furthermore, it has been shown that dose escalation using a 2-Gy per-fraction approach compared with a uniform dose of radiotherapy for all patients with concurrent chemotherapy provides no survival benefit and may in fact be detrimental.^27^ Additional treatment approaches that have been investigated but have failed to demonstrate a benefit over cCRT in patients with stage III NSCLC include the commonly used chemotherapy regimen pemetrexed–cisplatin combined with thoracic radiation, and maintenance treatment with the epidermal growth factor receptor (EGFR) inhibitor gefitinib following cCRT in an unselected population.^28,29^ As such, no significant advances in the treatment of patients with unresectable stage III NSCLC have been made over many years.^30^ The expanding role of immunotherapy in metastatic NSCLC, along with preclinical data suggesting that chemotherapy and radiotherapy upregulate PD-L1 expression on tumour cells, and may be synergistic with ICI,^18,31^ provides rationale for the evaluation of immune-checkpoint inhibitors and cCRT in the treatment of patients with earlier-stage NSCLC.

### PACIFIC trial: durvalumab (Imfinzi^®^▼, AstraZeneca UK Limited) following chemoradiotherapy in locally advanced, unresectable, stage III NSCLC

Durvalumab is a selective, high-affinity, human Ig-G1 kappa monoclonal antibody that blocks PD-L1 binding to PD-1 and CD80 (B7.1), allowing T cells to recognise and kill tumour cells.^32^ Early- phase clinical trials of durvalumab have shown promising results for patients with advanced solid tumours, including patients with stage III NSCLC.^33^

The Phase 3 PACIFIC trial randomised 713 patients with histologically or cytologically confirmed locally advanced, unresectable, stage III NSCLC who had completed cCRT, defined as two or more cycles of platinum-based chemotherapy concurrently with definitive radiotherapy (the use of consolidation chemotherapy was not permitted). Following confirmation of stable disease after cCRT, 709 patients received at least one dose of either durvalumab or placebo every 2 weeks for up to 12 months in a 2:1 ratio, respectively.^30^

**Fig. 1:**
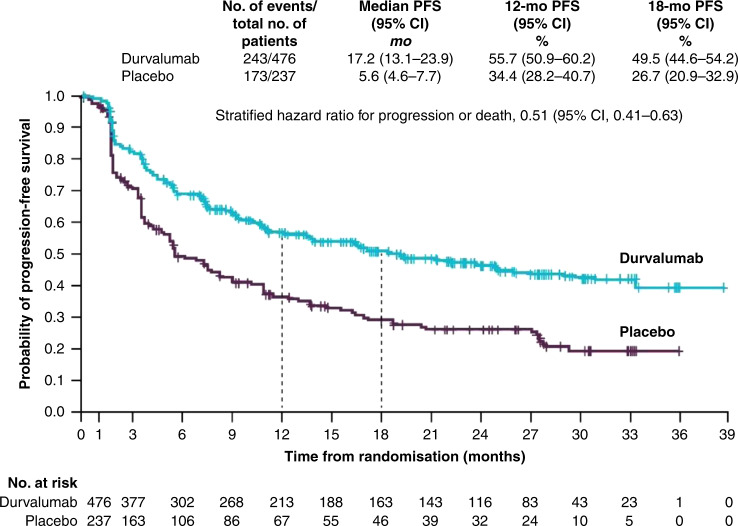
Updated progression-free survival (PFS)* for patients with unresectable stage III NSCLC who responded to prior chemoradiotherapy, receiving durvalumab or placebo in the PACIFIC clinical trial. Shown are Kaplan–Meier curves for updated PFS, defined according to the RECIST v1.1, and assessed by blinded-independent central review. Tick marks indicate censored observations, and vertical dotted lines indicate the times of landmark PFS analyses. The intention-to-treat population included all patients who underwent randomisation. Data cut-off for updated PFS was March 22, 2018, and median follow-up was 25.2 months. *No formal statistical comparison was made at this analysis for PFS because the study had achieved significance for PFS at the first planned interim analysis (data cut-off of Feb 13, 2017).^30^ Adapted from Antonia et al.^30^ Reprinted with permission from Massachusetts Medical Society.

Baseline characteristics were well-balanced between the two groups, including prior use of induction chemotherapy and initial response rates to cCRT. PD-L1 and EGFR status were measured using archival tissue rather than through mandatory biopsy samples, and differences between the groups were minimal. At the interim analysis (median follow-up of 14.5 months), durvalumab treatment resulted in a significantly longer PFS compared with placebo (16.8 vs. 5.6 months, respectively; HR: 0.52; 95% CI: 0.42–0.65; *P* < 0.001). The 12-month PFS rate was 55.9% versus 35.3%, and the 18-month rate was 44.2% versus 27.0% for the durvalumab and placebo arms, respectively. Subgroup analysis of prognostic factors, such as patient demographics, smoking status, clinical stage, histological subtype, response rate, PD-L1 and EGFR status all demonstrated PFS benefit with durvalumab. Favourable PFS, irrespective of PD-L1 expression and EGFR status, was of particular interest. Response rates were significantly higher following durvalumab treatment (28.4% vs. 16.0%; *P* < 0.001), and were shown to be durable with a longer median duration of response in the durvalumab treatment arm compared with placebo (not reached vs. 13.8 months; HR: 0.43). Ongoing response at 18 months was also in favour of durvalumab (72.8 vs. 46.8%, Fig. 1).

**Fig. 2:**
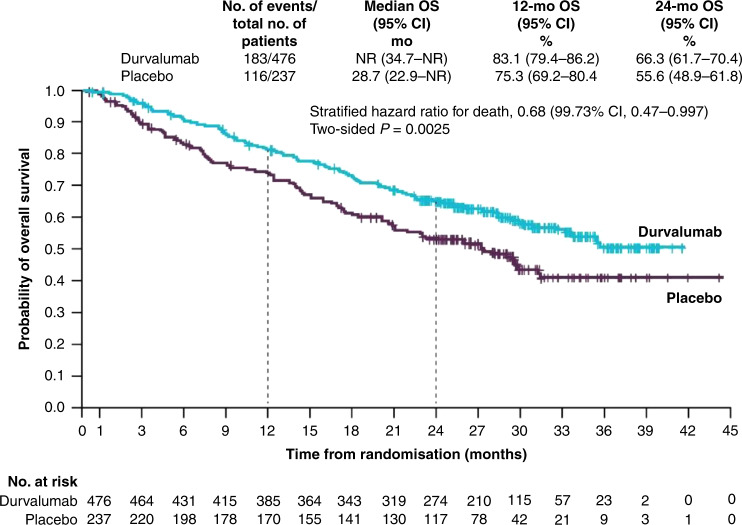
Median overall survival for patients with unresectable stage III NSCLC who responded to prior chemoradiotherapy, receiving durvalumab or placebo in the PACIFIC clinical trial. Shown are Kaplan–Meier curves for median OS, defined according to the RECIST v1.1, and assessed by blinded-independent central review. Tick marks indicate censored observations, and vertical dotted lines indicate the times of landmark OS analyses. The intention-to-treat population included all patients who underwent randomisation. Data cut-off for median OS was March 22, 2018, and median follow-up was 25.2 months. Adapted from Antonia et al.^34^ Reprinted with permission from Massachusetts Medical Society.

**Fig. 3:**
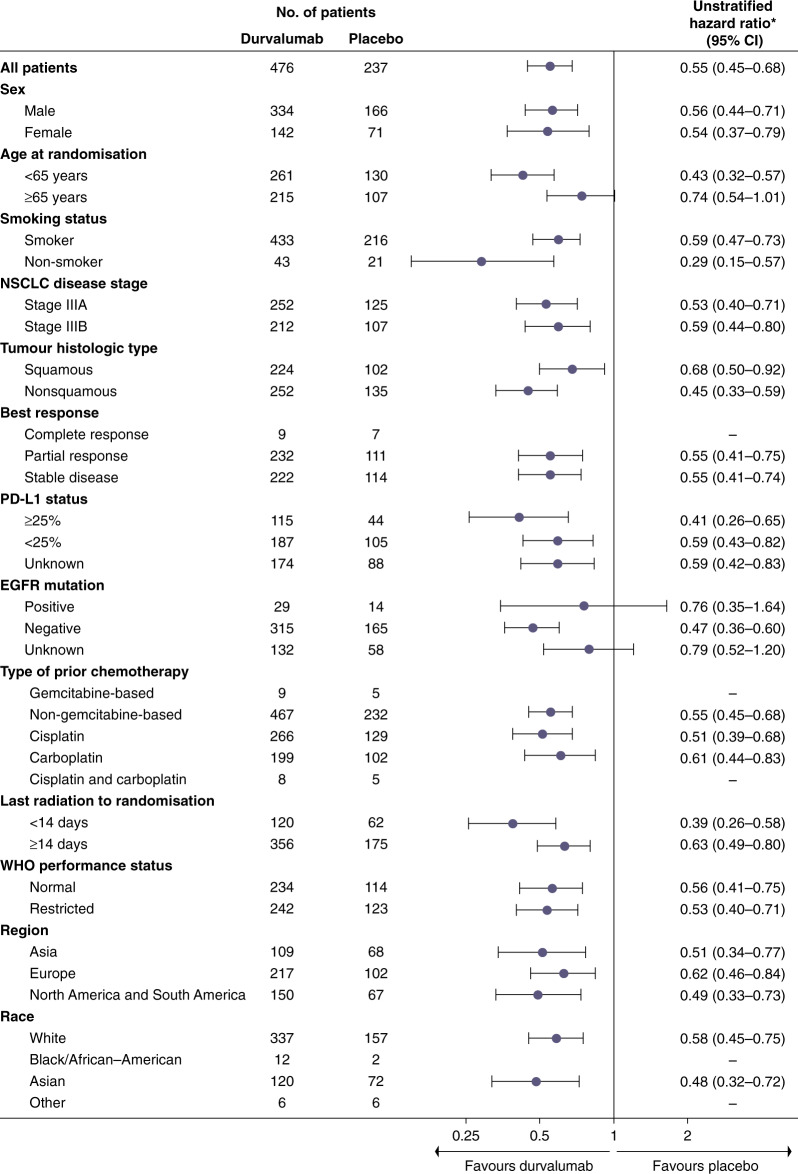
Subgroup analysis of prognostic and additional factors for progression-free survival (PFS) for patients in the intention-to-treat population of the PACIFIC trial. Shown are forest plots for subgroup analyses of prognostics and additional factors for PFS by pre-specified subgroups in the ITT population. PFS was defined according to the RECIST v1.1, and assessed by blinded-independent central review. The intention-to-treat population included all patients who underwent randomisation. Data cut-off for PFS was February 13, 2017, and median follow-up was 14.5 months. Hazard ratio and 95% confidence intervals were not calculated if the subgroup had <20 events. CT chemotherapy, HR hazard ratio, NA not available, NR not reached. OS overall survival, PFS progression-free survival. Adapted from Antonia et al.^30^ Reprinted with permission from Massachusetts Medical Society.

**Fig. 4:**
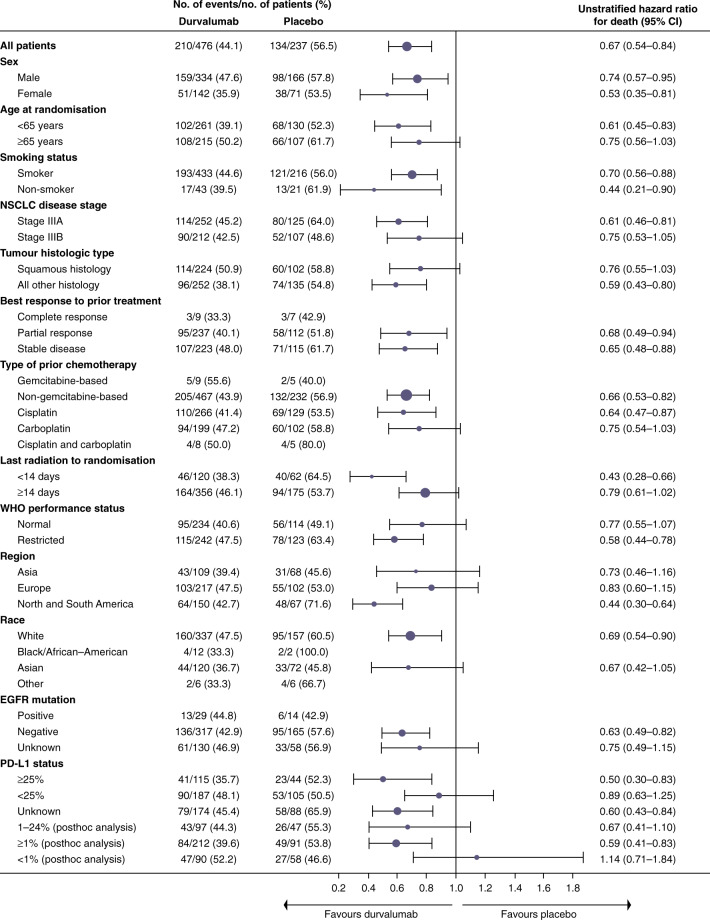
Subgroup analysis of prognostic and additional factors for exploratory 3-year overall survival for patients in the intention-to-treat population of the PACIFIC trial. Shown are forest plots for subgroup analyses of prognostic and additional factors for updated 3-year OS by pre-specified and post hoc exploratory subgroups in the ITT population. OS was defined according to the RECIST v1.1, and assessed by blinded-independent central review. The intention-to-treat population included all patients who underwent randomisation. Data cut-off for 3-year OS was January 31, 2019, and median follow-up was 33.3 months. Hazard ratio and 95% confidence intervals were not calculated if the subgroup had <20 events. CT chemotherapy, HR hazard ratio, NA not available, NR not reached, OS overall survival, PFS progression-free survival. Adapted from Gray et al.^34^ Copyright (2019), with permission from Elsevier.

**Fig. 5:**
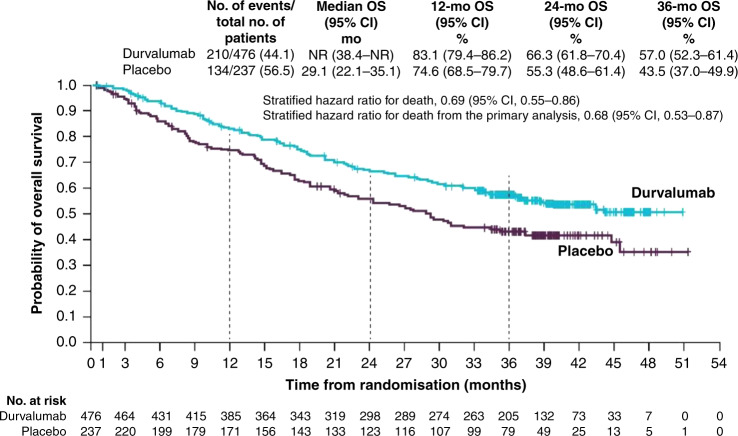
Exploratory 3-year overall survival for patients with unresectable stage III NSCLC who responded to prior chemoradiotherapy, receiving durvalumab or placebo in the PACIFIC clinical trial. Shown are Kaplan–Meier curves for post hoc, exploratory 3-year OS, defined according to the RECIST v1.1, and assessed by blinded-independent central review. Tick marks indicate censored observations, and vertical dotted lines indicate the times of landmark OS analyses. The intention-to-treat population included all patients who underwent randomisation. Data cut-off for 3-year OS was January 31, 2019, and median follow-up was 33.3 months. Adapted from Gray et al.^34^ Copyright (2019), with permission from Elsevier.

At the time of the final data cut-off, representing a median follow-up of 25.2 months, OS and an additional PFS analysis were performed. PFS was similar to the initial data cut-off (17.2 vs. 5.6 months; HR: 0.51; 95% CI: 0.41–0.63, Fig. 1), demonstrating a PFS benefit of 11.6 months in patients receiving durvalumab compared with those receiving placebo.^34^ Durvalumab significantly prolonged median OS (not reached vs. 28.7 months; HR: 0.68; 99.73% CI: 0.47–0.997; *P* = 0.0025, Fig. 2). Twenty-four month survival was 66.3% versus 55.6% in favour of durvalumab (*P* = 0.005). Like PFS, this survival benefit with durvalumab was seen across all the aforementioned subgroups (Figs. 3 and 4). Of particular interest is the reduction in brain metastases associated with durvalumab (6.3% vs. 11.8%). Durvalumab also extended the time to the first (HR: 0.58; 95% CI: 0.47–0.72) and second (HR: 0.63; 95% CI: 0.50–0.79) subsequent lines of treatments, as well as to second progression or death (HR: 0.58; 95% CI: 0.46–0.73) compared with placebo.^34^ A recently reported post hoc exploratory analysis of OS, performed after a median duration of follow-up of 33.3 months, was consistent with that previously reported with a 31% reduction in the risk of death (median not reached with durvalumab vs. 29.1 months with placebo; stratified HR 0.69; 95% CI: 0.55–0.86) and subgroup analyses of OS at this time, including by PD-L1 status, was consistent with those reported at the time of the primary OS analysis. The 12-, 24- and 36-month OS rates were all improved with durvalumab compared with placebo (83.1% vs. 74.6%, 66.3% vs. 55.3% and 57.0% vs. 43.5%, respectively, Fig. 5). In addition, consistent with the results reported at the time of the primary OS analysis, time to the first (HR: 0.58; 95% CI: 0.47–0.71) and second (HR: 0.61; 95% CI: 0.49–0.75) subsequent lines of treatments was markedly longer following durvalumab treatment compared with placebo.^35^

As of the time of the final data cut-off for OS (safety data were not collected at the post hoc analysis), adverse events (AEs) of any cause and grade were reported in slightly more patients who received durvalumab than placebo (96.8% vs. 94.9%, respectively), and similarly there was a difference in grade 3 or 4 AEs between treatment arms (30.5% vs. 26.1%).^34^ The most common grade 3 or 4 AE was pneumonia in both cohorts occurring in 4.4% of patients in the durvalumab arm and 3.8% in the placebo arm. Of the 15.4% of patients in the durvalumab arm and 9.8% of patients in the placebo arm who discontinued treatment, the commonest reason was pneumonitis (4.8% vs. 2.6%, respectively). Both radiation pneumonitis and pneumonitis due to other causes (expected following cCRT) were higher in patients who received durvalumab; however, grade 3 or 4 toxicity were both infrequent and similar in each treatment arm. As expected, there were higher numbers of patients reporting immune-related AEs of any grade in the durvalumab arm; however, this was in the range that would be expected from other studies using immune-checkpoint inhibitors.^8,12,34^

The design of the PACIFIC trial had a number of limitations, including the wide range of chemotherapy regimens permitted for cCRT (including platinum with etoposide, vinblastine, vinorelbine, paclitaxel, docetaxel or pemetrexed). Although this range of regimens reflects clinical practice, it may contribute to difficulties in interpreting the study data. In addition, patients were eligible for randomisation into the study between 1 and 42 days following completion of cCRT (allowing for resolution of acute toxic effects associated with cCRT). This is a different approach to other historical randomised studies exploring experimental approaches with cCRT. In the comparable patient population of the RTOG 0617 trial, PFS in the control cCRT group was 11.8 months (from initiation of CRT), and in the START trial, PFS in the control group was 8.4 months (from completion of CRT).^27,36^ However, in the PACIFIC study, PFS was measured from the time of randomisation that could occur up to 42 days after completion of cCRT. Factoring in these variabilities in trial design, the PACIFIC control group behaved similarly to control groups in these previous Phase 3 trials.^37^ In addition, patients had to have stable disease following completion of cCRT to be eligible for randomisation into the PACIFIC trial. Of 983 patients who were enrolled, 270 were not randomised, of which 225 did not meet study criteria for inclusion. The reasons behind exclusion from randomisation in this group remain unclear.

These compelling survival data from the PACIFIC study led to durvalumab receiving marketing authorisation from the European Medicines Agency (EMA) in October 2018 as monotherapy for the treatment of locally advanced, unresectable NSCLC in adults whose tumours express PD-L1 on ≥1% of tumour cells, and whose disease had not progressed following platinum-based CRT.^38^ Although the PACIFIC trial met its primary PFS endpoint both in the overall patient population and in all 35 pre-specified subgroups, the EMA has restricted the European indication to only those patients who are PD-L1 positive (≥1% PD-L1), which is based on an exploratory post hoc analysis.

When the PACIFIC study was initiated, no predictive biomarker for immunotherapy had been fully validated, and tissue collection at diagnosis was not mandatory. An exploratory analysis of PD-L1 expression on tumour cells from biopsies before CRT was prespecified, with a planned cut-off of 25% (but no stratification by PD-L1 expression performed), and notably, the PFS benefit with durvalumab was observed, irrespective of PD-L1 expression (HR: 0.59; 95% CI: 0.43–0.82 for a PD-L1 expression level of <25% and HR: 0.41; 95% CI: 0.26–0.65 for a PD-L1 expression level of ≥25%).^30^ An exploratory post hoc analysis of PFS by PD-L1 expression levels (<1%, ≥1%, 1–24% and ≥25%) on tumour cells at initial biopsy was requested by the EMA. In this unplanned analysis, the beneficial effect of durvalumab on PFS was consistent across PD-L1 expression groups. The OS benefit was consistent in the PD-L1 unknown group and the overall population, but an OS benefit was not observed in the PD-L1-negative (<1%) subgroup.^38^ There are mixed views on the decision by the EMA to restrict durvalumab treatment to patients with tumours with PD-L1 expression ≥1%, with some believing that further investigation is warranted in this population,^39^ whereas others believe that the EMA should have approved durvalumab based on the pre-specified intention-to-treat population, rather than on an exploratory post hoc analysis that was not powered to show a statistically significant difference between treatment arms.^40^

The inclusion criteria for the PACIFIC trial stated that patients had to have completed cCRT, defined as two or more cycles of platinum-based chemotherapy concurrently with definitive radiotherapy. Although considered the global gold standard of care for fit patients with stage III NSCLC, this treatment has not been adopted as standard practice in all cancer centres. The additional value of durvalumab following cCRT demonstrated in the PACIFIC study reinforces the importance for all centres to adopt cCRT algorithms for appropriate patients to ensure that they have the opportunity to gain maximum benefit from this multi-modality therapeutic approach.

### Further studies of immunotherapy for stage III NSCLC

#### Studies of immunotherapy following cCRT for stage III NSCLC

There are numerous studies ongoing and under development further evaluating the optimal way of combining ICI with cCRT, although only durvalumab is licensed in stage III after completion of cCRT.

Nivolumab was being investigated in a randomised controlled Phase 3 trial following cCRT (RTOG 3505; NCT02768558); however, this study was terminated in February 2019 because other treatments have been found to be efficacious,^41^ whilst pembrolizumab following cCRT demonstrated promising efficacy in an open-label Phase 2 trial in the same setting (LUN 14-179; NCT02343952).^42^ Patients in both trials require histologically or cytologically confirmed, unresectable stage III NSCLC, with no progression after cCRT.

Within the RTOG 3505 trial, patients received 60 Gy of radiotherapy with concurrent cisplatin and etoposide chemotherapy, and upon completion of cCRT, patients were randomised 1:1 to receive placebo or nivolumab (every 2 weeks) for up to 12 months, with treatment commencing 4–12 weeks after completion of cCRT. Primary endpoints included OS and PFS.^41^ Within the LUN 14-179 Phase 2 trial, patients received 59.4–66.6 Gy of radiotherapy with concurrent cisplatin/etoposide, carboplatin/paclitaxel or cisplatin/pemetrexed chemotherapy, with all patients receiving pembrolizumab (every 3 weeks) for up to 12 months, starting within 28–56 days after completion of cCRT.^43^ Early results of the LUN 14-179 trial (after enrolment of 93 patients and a median follow-up of 16.4 months) are comparable to that of the PACIFIC trial,^30^ with a median PFS of 15.4 months (95% CI 10.4–NR) and 12-, 18- and 24-month PFS rates of 59.9%, 49.5% and 45.4%, respectively. There was one death related to pneumonitis and five (5.4%) patients had grade 3–4 pneumonitis.^43^ Updated PFS and the final OS results from the LUN 14-179 study are eagerly awaited and may provide further evidence for the use of immunotherapy following the completion of cCRT in patients with stage III NSCLC.

#### Studies of immunotherapy in stage III NSCLC

The timing of when to deliver ICI treatment following cCRT may play a significant role in the benefits patients receive from this treatment regimen. In the PACIFIC study, both PFS and OS were greater in patients who commenced durvalumab treatment within 14 days following completion of cCRT, compared with those who received treatment ≥14 days (Figs. 3 and 4).^30^ This finding should be interpreted with caution as it may be as a result of selection bias, with patients initiating treatment within 14 days potentially being able to start treatment earlier due to improved fitness or smaller volumes compared to those starting treatment after 14 days.

The optimal duration of treatment with ICIs following cCRT is also currently unknown; the PACIFIC, RTOG 3505 and the LUN 14-179 studies offer(ed) up to 12 months of treatment; however, no studies have compared different treatment durations within this setting. Therefore, further studies are required to assess the optimal duration of immunotherapy following cCRT.

### Considerations for obtaining optimal responses to immunotherapy in NSCLC

In addition to treatment sequence, appropriate patient selection is crucial to ensure maximal benefit without undue risk.^44^ Improved understanding of immune biology has led to the discovery of further predictive biomarkers of response beyond PD-L1 expression, including tumour mutational burden (TMB)^13,45^ and immune gene signatures.^14^ Challenges faced with these biomarkers include adequate tissue sampling, a robust reproducible diagnostic assay, intra-tumoural heterogeneity of TMB and PD-L1 expression and inter-tumoural heterogeneity in patterns of response to ICIs.^46^ Next-generation sequencing of circulating tumour DNA may provide a non-invasive approach to identifying predictive biomarkers, such as TMB in the future.^47^

Striking a balance between immune toxicity and predicting response to ICI treatment is crucial in sparing patients undue side effects whilst taking pressure off an already-fraught United Kingdom (UK) health economy.^48^ In addition, predicting patients who may experience resistance to ICI treatment and disease progression following an initial response requires further investigation. Understanding the mechanisms of resistance and changes in biomarker expression in response to treatments may guide future rationale for treatment choices.

The mechanisms of resistance to ICIs remain a key area of research, which is being extensively studied to better understand the reasons for poor clinical response. Potential mechanisms explored include immune-mediated resistance such as T-cell exhaustion, poor generation of T-cell memory and insufficient antitumour T cells^49^ or tumour-mediated resistance, such as genomic alterations in STK11/LKB1, which modulates the tumour microenvironment to downregulate PD-L1 expression.^50,51^ Developing techniques to overcome these obstructions to ICI sensitivity is critical for improving both primary resistance and the durability of response.

### Future considerations for immunotherapy in stage III NSCLC

The PACIFIC study explored a patient population with locally advanced NSCLC treated with cCRT; however, it did not include patients not considered fit to receive this intensive regimen, including those treated by sequential chemoradiation, chemotherapy or radiation alone. Other challenging patient populations include those with resected stage III NSCLC who may or may not have residual mediastinal lymphadenopathy.

The PACIFIC study suggested that patients with EGFR mutations also conferred an improvement in PFS (HR: 0.76), although this was less than in patients without an EGFR mutation (HR: 0.47) and patient numbers within the EGFR mutation subgroup were small (*n* = 29 and *n* = 14 for the durvalumab and placebo arms, respectively). Additional studies to determine the benefit of immunotherapy in these patients would be of interest.^30^

Alternative treatment strategies for combining radiotherapy and immunotherapy have also been proposed, potentially for patients who may not be able to tolerate chemotherapy. However, only if radiotherapy is delivered to as much of the tumour burden as can be safely irradiated, enabling infiltration of the radiation-primed cells into all parts of all tumours, is a successful systemic response following ICI likely.^52^ Additionally, sequencing of radiotherapy and immunotherapy (combined or separately) may also be important to their combined efficacy.^52^

## Conclusions

The key data from the Phase 3 PACIFIC study in patients with documented locally advanced, unresectable stage III NSCLC, described here, demonstrate the clinical efficacy and long-term clinical benefit of durvalumab in this setting with significant improvements in all efficacy endpoints evaluated (OS, PFS and objective response rate) compared with placebo.^35^ These survival benefits, along with a tolerable safety profile, have led to durvalumab receiving marketing authorisation from the FDA for the treatment of patients with unresectable non-small-cell lung cancer that has not progressed after chemoradiation. This was followed by the EMA, which recognised the clinical benefit of durvalumab in adults with stable unresectable stage III NSCLC following two or more cycles of platinum-based cCRT, whose tumours express PD-L1 on ≥1% of tumour.^38^

Pembrolizumab and nivolumab are approved for the treatment of patients with stage IV NSCLC, and are being investigated in ongoing Phase 2 and 3 trials, in patients with stage III disease. Additional considerations are required to determine the optimal use of immunotherapy following cCRT, including duration of treatment and the exploration and identification of predictive biomarkers of response. It remains important to routinely assess the PD-L1 status in stage III NSCLC at diagnosis, to ensure that we offer patients the opportunity to gain the potential benefits of this multi-modality approach, with curative intent as the main goal. Although the survival analysis from PACIFIC remains immature, we eagerly await further long-term survival updates. Four-year survival data from clinical trials of adjuvant immunotherapy in other tumour types, such as advanced malignant melanoma, suggest a durable, sustained survival benefit; however, whether this equates to a cure remains to be seen.^53^

This patient selection strategy should be coupled with appropriate management strategies throughout the entire journey to ensure that patients have the highest physiological reserve and a positive outlook, to gain the benefits of the significant advancements in the standard of care for patients with stage III NSCLC.

## Data Availability

Not applicable.

## Appendix

**PRESCRIBING INFORMATION**

**IMFINZI**^®^ **▼(durvalumab) 50** **mg/ml solution for infusion**

https://medicines.astrazeneca.co.uk/content/dam/multibrand/uk/en/prescribinginformation/imfinzi-pi.pdf
